# Draft genome sequences of *Lactiplantibacillus plantarum* strains KABP062 and KABP063 isolated from human feces

**DOI:** 10.1128/mra.00196-26

**Published:** 2026-04-20

**Authors:** Pol Huedo, Erola Astó, Marta Perez, Maria Rodriguez-Palmero, Jordi Espadaler-Mazo

**Affiliations:** 1 R&D Department, AB-Biotics S.A. (Part of Kaneka Corporation) 602131 , Barcelona, Spain; 2 Basic Sciences Department, Universitat Internacional de Catalunya 16760, Barcelona, Spain; Wellesley College, Wellesley, Massachusetts, USA

**Keywords:** probiotics, microbiota, microbiome, genomics

## Abstract

We present the draft genome sequences of *Lactiplantibacillus plantarum* strains KABP062 and KABP063, two fecal isolates with probiotic potential.

## ANNOUNCEMENT

*Lactiplantibacillus plantarum* is a metabolically versatile, nomadic lactic acid bacterium with relevance in the food industry and human health (1). Genome sequencing of novel *L. plantarum* strains is essential to explore their probiotic potential.

*L. plantarum* strains KABP062 and KABP063 were isolated in 2005 from fecal samples from two different healthy Peruvian kids (Cajamarca, Perú, 7°9′49.6″ S, 78°30′1″ W), whose legal tutors gave informed, explicit consent. Fresh fecal samples were diluted in phosphate-buffered saline and seeded onto de Man, Rogosa, and Sharpe (MRS) agar plates and incubated at 37°C for 48 h in anaerobic jars. Bacterial species were firstly identified through 16S rDNA sequencing using primers 27F (5′-AGAGTTTGATCMTGGCTCAG-3′) and 1492R (5′-TACGGYTACCTTGTTACGACTT-3′) (2), amplicons being queried against the 16S RNA sequences database in BLAST (3). Genomic average nucleotide identity was verified with JSpecies (4). Antibiotic susceptibility was performed by the broth microdilution method following ISO 10932:2010 (5), as recommended by EFSA (6).

Isolated colonies were inoculated into 10 mL of MRS medium and cultured as indicated above. Cultures were centrifuged, and genomic DNA was extracted from pellets using the QIAamp DNA Blood Mini Kit (Qiagen, USA) following manufacturer instructions. Whole-genome sequences were obtained at Macrogen (Korea). Genome libraries were prepared by Nextera XT DNA Preparation kit and sequenced by Illumina NovaSeq 6000 generating 2 × 150 bp paired-end reads. Trimmomatic v0.38 (7) was used to remove adapter sequences and low-quality reads. *De novo* assembly was performed using SPAdes v3.15.0 (8). The assembled genomes were validated using BWA-MEM v0.7.17-r1188 aligner (9) and BUSCO v5.1.3 analysis (10). Annotation was performed through NCBI Prokaryotic Genome Annotation Pipeline v6.7 (11). Default parameters were used except where otherwise noted.

Results of genome sequencing are shown in Table 1. Both strains KABP062 and KABP063 showed MICs below EFSA cut-off values for *L. plantarum* (6). Specifically, they exhibited 0.5 µg/mL for ampicillin, 4 µg/mL for gentamicin, 16 µg/mL for kanamycin, 0.5 µg/mL (KABP062) and 0.25 µg/mL (KABP063) for erythromycin, 0.5 µg/mL for clindamycin, 8 µg/mL (KABP062) and 4 µg/mL (KABP063) for tetracycline, and 4 µg/mL (KABP062) and 2 µg/mL (KABP063) for chloramphenicol.

**TABLE 1:** Genome assembly, annotation, and quality metrics of *Lp^a^* strains KABP062 and KABP063

Feature	*Lp* KABP062	*Lp* KABP063
Total reads	17,856,196	20,853,774
Number of contigs	53	38
Genome size (bp)	3,262,171	3,251,349
N50 (bp)	130,217	247,484
Depth	175×	190×
G + C content (%)	44.5	44.4
BUSCO completeness	98.39% complete (122 C, 2 D, 0 F, 0 M; 124 total)	98.39% complete (122 C, 2 D, 0 F, 0 M; 124 total)
Total genes (Prokaryotic Genome Annotation Pipeline)	3,152	3,149
Protein-coding sequences	3,020	3,011
tRNA genes	66	66
5S rRNA	5	6
16S rRNA	3	1
23S rRNA	1	1
16S rRNA sequencing best hit (%)	99.93 (*Lp* JCM 1149)	99.93 (*Lp* JCM 1149)
Average nucleotide identity of *L. plantarum* ATCC 14917 (%)	98.83	98.83

^*a*^ *Lp*, *Lactiplantibacillus plantarum*.

Further *in vitro* and clinical validation will be required to confirm probiotic activity of *L. plantarum* strains KABP062 and KABP063.

## Data Availability

The whole-genome sequencing project for *L. plantarum* KABP062 has been deposited at GenBank under accession number JBUPYW000000000. The raw sequencing data are available in the Sequence Read Archive (SRA) database under accession number SRR37084844. The whole-genome sequencing project for *L. plantarum* KABP063 has been deposited at GenBank under accession number JBUPYV000000000. The raw sequencing data are available in the SRA database under accession number SRR37084843. The associated BioProject accession number is PRJNA1418146.
